# A pilot cluster randomised controlled trial to investigate the addition of direct access to physiotherapy to usual GP-led primary care for adults with musculoskeletal pain: the STEMS pilot trial protocol (ISRCTN23378642)

**DOI:** 10.1186/s40814-015-0020-4

**Published:** 2015-07-17

**Authors:** Annette Bishop, Stephanie Tooth, Joanne Protheroe, Chris Salisbury, Reuben O Ogollah, Sue Jowett, Elaine M Hay, Nadine E Foster, on behalf of the STEMS study team

**Affiliations:** 1 Research Institute for Primary Care & Health Sciences, Keele University, Keele, Staffordshire ST5 5BG UK; 2 Centre for Academic Primary Care, University of Bristol, Bristol, UK; 3 Health Economics Unit, University of Birmingham, Birmingham, UK

**Keywords:** Physiotherapy, Direct access, Self-referral, Musculoskeletal, Cluster trial, Pilot

## Abstract

**Background:**

Musculoskeletal problems are common, accounting for up to 30 % of general practitioner (GP) consultations and are a major cause of chronic disability worldwide. Demand for health care for musculoskeletal conditions is likely to continue to rise given the ageing population and the increasing impact of these common painful conditions. Physiotherapists are well equipped to deliver evidence-based management for these conditions. Direct access allows patients to access physiotherapy without seeing their GP or another referring practitioner first; however, for most patients in the UK, access to National Health Service physiotherapy is controlled through GP referral.

**Methods/Design:**

The aim of this pilot, pragmatic, cluster trial is to assess the feasibility of a future large trial to compare the clinical and cost-effectiveness of the additional offer of direct access to physiotherapy versus continuing with usual GP-led primary care alone for adults with common musculoskeletal problems. The pilot will focus on process outcomes to assess feasibility, although performance of the likely outcomes of a main trial will also be assessed. This is a two-arm parallel, cluster RCT where GP practices are the units of randomisation (the clusters), yet data are collected from individual patients with musculoskeletal problems (the participants). A direct access service will be set up in the participating physiotherapy service to provide the option of direct access to patients of the intervention arm practices. Inclusion criteria are broad to reflect the ‘real-world’ operation of an NHS physiotherapy direct access service for patients with musculoskeletal pain. Data collection will be through patient self-reported questionnaires at baseline, 2, 6 and 12 months and medical record review.

**Discussion:**

No previous trials have been conducted into direct access to physiotherapy for patients with musculoskeletal problems. The strengths of the STEMS pilot trial are its size, the length of follow-up, and collection of process, clinical and cost outcomes to fully inform a future main trial to meet calls to provide robust trial evidence of the impact on clinical outcomes, work loss and costs to provide clinicians and service funders with the high quality trial data they need to guide decisions on the best models of care.

**Trial registration:**

The STEMS pilot trial is registered at Current Controlled Trials: ISRCTN23378642

**Electronic supplementary material:**

The online version of this article (doi:10.1186/s40814-015-0020-4) contains supplementary material, which is available to authorized users.

## Background

Musculoskeletal problems are common and costly and increase with age. Musculoskeletal conditions account for up to 30 % of general practitioner (GP) consultations [1], and yet it is estimated that only between 30–40 % of individuals with musculoskeletal problems consult their GP [2–4]. The Global Burden of Disease Study shows that musculoskeletal conditions are a major cause of chronic disability worldwide and have increased markedly in the last decade [5]. Given the ageing population and the increasing impact of these common painful conditions, the demand for musculoskeletal healthcare is set to rise and health care delivery systems will need to develop a ‘coherent policy for dealing with musculoskeletal disorders’ [5]. Best evidence for many of these musculoskeletal conditions recommends treatments such as advice, education, exercise, manual therapy and acupuncture [1, 6, 7]. Such treatments are cost-effective [8] and are those that professionals such as physiotherapists are equipped to deliver. Early physiotherapy intervention for musculoskeletal problems reduces sick leave and helps prevent acute problems becoming chronic [1, 9–12]. However, for most patients in the UK, access to National Health Service (NHS) physiotherapy is controlled through GP referral.

Direct access to physiotherapy is a system of access in which ‘patients are able to refer themselves to a physiotherapist directly without having to see anyone else first or without being told to refer themselves by a health professional’ [13]. Although direct access is well established in private practice in the UK and in other countries including Australia, the Netherlands, some states within the USA and in Scotland [14–17], uptake in the NHS in England has been very limited. Funders of physiotherapy services express concerns about creating excessive demand and associated costs and where direct access services have been established at least one in England has been stopped due to funding being withdrawn [18].

To date, evidence about direct access to physiotherapy comes from observational studies only. These have suggested that direct access may reduce GP workload [19] with estimates of a 20 % reduction in multiple GP consultations [20]. An evaluation in the Netherlands reported that in the first 12 months of direct access, patients referring themselves accounted for 22–28 % of all physiotherapy referrals [14, 21]. Reported patient benefits of direct access are greater freedom of choice and improved access to musculoskeletal care [13, 14, 19, 22], since direct access allows the patient to opt for physiotherapy when they are in most need. There are also some suggested cost-benefits, with GP referral costing £133 an episode and an episode of care through direct access costing £100 [23].

A global review of direct access to physiotherapy has identified professional legislation, the medical profession, politicians and policy makers as both barriers to and facilitators of direct access [24]. A European-focused review of direct access services concluded that clinicians, managers and service funders need high quality trial data on both clinical and cost-effectiveness to guide decisions on the best models of care [25]. In addition, an independent evaluation of the observational UK Department of Health pilots of direct access to physiotherapy [13] concluded that robust evidence of the impact on clinical outcomes, work loss and costs are lacking due to important flaws in observational study designs and that a randomized controlled trial (RCT) comparing areas that do and do not offer direct access is required to provide robust evidence about direct access [26].

The most appropriate design for a RCT to evaluate direct access is a non-inferiority cluster RCT that compares the clinical and cost-effectiveness of the additional offer of direct access to physiotherapy versus continuing with usual GP-led care alone for patients with musculoskeletal conditions in primary care. However, given that this involves developing a new direct access service, it is important to conduct feasibility and pilot work first in order to fully inform a future main trial.

### Overall aim

The overall aim of the STEMS pilot trial is to assess the feasibility of a future large trial to compare the clinical and cost-effectiveness of the additional offer of direct access to physiotherapy versus continuing with usual GP-led primary care alone for adults with common musculoskeletal problems.

### Objectives

The objectives of the STEMS pilot trial include both process and research objectives. Analysis of the process objectives will enable the feasibility of a larger cluster RCT to be assessed. They focus on the feasibility of working with general practices and physiotherapy services to develop and set up a direct access to physiotherapy service, the acceptability of direct access and the ability to both recruit and retain participants in the research evaluation.

#### Process objectives

1. To assess the number of practices approached and agreeing to take part (baseline) and the engagement of GP practices and physiotherapy services to stay in the pilot trial through follow-up (12 months)
2. To develop and test approaches to market a new direct access service in ways that ensure a sufficient proportion (at least 20 %) of patients access physiotherapy through self-referral to make a main trial feasible (assessed through referral methods as physiotherapy service)
3. To assess the feasibility of establishing a physiotherapy direct access service that can respond to demand and avoid increases in waiting times or staffing levels (monitored throughout the recruitment period)
4. To estimate participant recruitment rates in both control and intervention practices (assessed at end of recruitment)
5. To explore any evidence of selection bias in participants recruited to the research evaluation from the control and intervention practices (assessed through participant characteristics at baseline)
6. To estimate retention of participants in the research evaluation at each follow-up time-point across both control and intervention practices

#### Research objectives

The pilot trial will also provide a useful test of outcome data collection methods, including key clinical outcomes, and provide information on the likely changes in these outcomes in the control and intervention groups. Whilst a main trial will provide the definitive test of the difference between trial groups on these clinical outcomes, the pilot has the following research objectives:

1. To investigate likely changes in the primary clinical outcome measure (physical health measured using the SF36v2 Physical Component Summary)
2. To investigate likely changes in secondary outcome measures (overall perceived change, mental health, pain self-efficacy, quality of life, understanding of the condition, experience of care and convenience and accessibility of service)
3. To provide an early estimate of the costs, both healthcare and societal costs, in both intervention and control groups
4. To explore the use of willingness to pay (WTP) methodology to capture the strength of patient preferences for direct access to NHS physiotherapy
5. To confirm the parameters needed for a realistic sample size calculation for a future main cluster RCT

## Methods

### Design

The design is a pilot, pragmatic, cluster RCT in general practice and physiotherapy services. The most appropriate trial design is a simple, two-arm parallel, cluster RCT where GP practices are the units of randomisation (the clusters), yet data are collected from individual patients with musculoskeletal problems (the participants). This design overcomes the problem of contamination between arms and the problems associated with individually consenting and randomising patients to a trial testing service-level changes. A main trial would employ a non-inferiority design as it will be essential that the intervention is as least as good as usual GP-led care in terms of patients’ clinical outcomes (physical health measured using the SF36v2 Physical Component Summary).

A repeated measures design will be adopted, whereby participants will complete the questionnaires at baseline, 2, 6 and 12 months. Each participant’s involvement in the trial is for 12 months, during which time they will all have access to usual GP-led primary care. No analysis of clinical outcomes will be undertaken until the 12-month follow-up time-point.

### Setting and clusters

The setting is primary care in England for adults with musculoskeletal problems (GP practices and linked NHS physiotherapy services). GP practices are the unit of randomisation, and thus, patients follow the care pathways to which the practice is randomised. In this pilot, RCT practices will be stratified only on practice size, but in a main trial, practices would also ideally be stratified by index of deprivation. Randomisation of GP practices will be undertaken by an independent statistician. GP practices will be randomised to one of two arms, either to continue with usual GP-led primary care for musculoskeletal patients with the addition of the offer of a self-referral physiotherapy pathway (intervention group) or to continue with usual GP-led primary care alone (usual care control group). Practices will be randomised according to a computer-generated random numbers stratified by the practice size (small or large) in the ratio 1:1. In a main study, we would plan to further stratify by practice setting and area level deprivation (through minimisation) in order to ensure balance in key practice characteristics in each arm of the trial.

GP practices are eligible to take part if they meet the following criteria: they are within the Vale Royal Clinical Commissioning Group area of Cheshire and the Clinical Research Network (CRN); they are a group practice, currently referring musculoskeletal patients to NHS physiotherapy services; they do not currently offer direct access to physiotherapy; and they are willing to test direct access. The balance between scientific considerations and the need for consent is a known issue for cluster trials [27]. Informed consent for practices to participate will be provided by the senior GP partner in each practice acting as ‘guardian’ for patients in their care. Patients will follow the care to which their practice is randomised with identical patient information for both arms providing general information about the study, explaining that their local musculoskeletal services are being evaluated using patient self-reported clinical outcomes and medical record review. Individual patients will therefore be able to opt out of data collection.

### Participants

The inclusion criteria have been designed to be as broad as possible to reflect the ‘real-world’ operation of an NHS physiotherapy direct access service for patients with musculoskeletal pain.

Inclusion criteria are all adults consulting participating GP practices or physiotherapy services with musculoskeletal problems (for either their first consultation for a new episode of musculoskeletal pain or a reconsultation).

Exclusion criteria are: under 18 years old at the time of consultation; consulting with non-musculoskeletal problems; unable to provide their own consent to the research evaluation; undergoing palliative care; severe learning disabilities; housebound or in nursing home accommodation; and unable to communicate in English (although all potential participants will be offered the opportunity to telephone a research nurse, blinded to practice allocation, for help in completing the paperwork).

### Participant identification and recruitment

Following randomisation of GP practices, potentially eligible participants will be identified through one of three methods.

Method 1: Patients consulting GP practices with a musculoskeletal problem will have a musculoskeletal Read code^1^ (entered by GPs or nurse practitioners) entered into their computerised medical record. A member of the GP practice team, or CRN staff, will download the details of patients with these Read codes twice weekly checking for exclusion criteria and then forward these details to a CRN administrator. GPs will be able to exclude any patients they perceive as being particularly vulnerable and unsuitable to approach for involvement in research by using a dedicated exclusion code set up for the study.

Method 2: In addition, for those GP practices randomised to also offer direct access to physiotherapy, patients who self-refer to physiotherapy will be identified when they forward a completed self-referral form to the physiotherapy service or telephone the physiotherapy administrator for help in completing a form. As in method 1 the details of identified patients will be forwarded to a CRN administrator.

Method 3: Patients referred by GPs or practice nurses to the physiotherapy service, but not identified in method 1 due to an absence of an appropriate Read code in the computerised medical records, will be identified from the physiotherapy administration database. A physiotherapy administrator will download the details of all patients referred from the participating GP practices twice weekly and forward these details to a CRN administrator. Duplication checks will ensure that eligible patients are not invited to take part in the research more than once.

All eligible patients will be mailed a STEMS study pack (letter of invitation, participant information leaflet (see Additional file 1), consent form, baseline questionnaire and pre-paid return envelope). Participants will not be individually consented to randomisation, rather participants in both arms of the trial will be asked to give written consent to take part in a study investigating musculoskeletal problems and local health services, consisting of the baseline and follow-up questionnaires (at 2, 6 and 12 months) and to allow the research team access to their medical records to review healthcare use for their musculoskeletal problem. Patients interested in participating will be offered the option of telephoning a research nurse, blinded to practice allocation, who will answer any questions and support those who need it to complete the consent form and questionnaire over the phone. Participants who consent to participation will return the completed questionnaire and consent form to the research centre, and the research team will then have access to their personal identification details. Participants completing the consent form but having missing data at baseline on the primary outcome measure (the SF36v2), such that a Physical Component Summary score cannot be calculated, will be contacted by telephone to collect the missing data. At each data collection time-point (baseline, 2, 6 and 12 months), non-responders will be mailed a postcard reminder 2 weeks after mailing of the trial study pack and a repeat study pack 2 weeks after the reminder postcard. At 6- and 12-month follow-up, non-responders will subsequently be mailed a very brief minimum data questionnaire, consisting of the SF-36v2 and the single Global Assessment of Change question only. Telephone collection of minimum data will be attempted for the remaining non-responders.

**Fig. 1:**
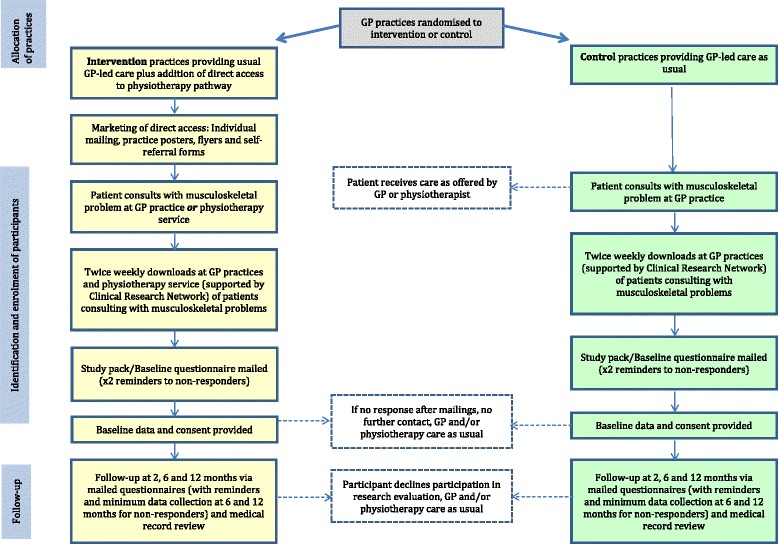
Flow chart of the STEMS pilot cluster randomised trial

This method of recruitment has been used successfully in previous studies. Our processes ensure that all eligible patients will be identified, recruited through postal study packs (with telephone support where needed) with identical information about the evaluation study given to those in both arms of the trial [27]. Participants’ GPs will be notified of their consent to participate in the research evaluation. Participating general practices will be supported to assist with identification of potentially eligible participants for the STEMS pilot trial through small practice payments to reimburse their time for screening patient lists. The physiotherapy service will be supported to participate through financial reimbursement for the time taken from service delivery for participation in the training programme. Participants will not receive any payments or other incentives to take part in the STEMS pilot trial or to return baseline or follow-up questionnaires. A flowchart illustrating the STEMS pilot trial is shown in Fig. 1.

### Methods for protecting against sources of bias

Allocation concealment for participating GPs and physiotherapists is not possible, and therefore, to prevent recruitment bias, neither of these groups of clinicians will be involved in recruiting participants into the research evaluation. Instead eligible participants will be identified from electronic GP medical records and from the physiotherapy service database, conducted by CRN staff. We will follow the CONSORT guidelines for both cluster trials and non-inferiority trials [28, 29]. In an ideal world, the comparison would be all patients who have a musculoskeletal problem in the participating practices during the study period, whether or not they attend their GP and/or physiotherapy service, but clearly this is not possible. Therefore, all patients who consult their GP practice (control arm) will be compared with all patients who consult their GP or physiotherapy service (intervention arm) with a musculoskeletal condition. Patients who directly access physiotherapy may be different in important ways to those who consult their GP, which is a limitation of the design. Therefore, in the pilot trial, we will investigate whether the number of consulters (to GPs only in the control arm and to either GPs or physiotherapists in the intervention arm) that have been identified in each arm are similar, whether there appears to be any differential uptake to the research evaluation in the two arms and whether the baseline characteristics of participants in each arm are similar.

Both primary and secondary outcomes are based on self-report postal questionnaires; therefore, no investigator bias will be introduced at assessment. The research nurses involved in helping patients to complete questionnaires and minimum data collection will be based in the Clinical Research Network, not in participating practices, and will be kept blind to practice allocation. Success of blinding will be recorded on the Minimum Data Collection form. In addition, the trial statistician undertaking the analysis will also be blind to practice allocation. Using validated outcome measures for self-report will guard against measurement error. Quality control including data entry, coding, security, storage and management will be performed according to the Standard Operating Procedures of the Keele Clinical Trials Unit. A random 10 % of the participants’ data entered will be compared with the paper versions to check data entry accuracy. Data accuracy will be audited and accuracy rates recorded. Loss to follow-up will be minimised by implementing standardised reminder procedures and minimal data collection for non-responders. Participants will be free to withdraw from the research at any time without having to give any explanation. Where possible, we will collect information about the reasons for withdrawal. All records will be kept confidential and data sets for each participant will be identified by the patient’s participant number. Analysis will be performed on an intention-to-treat (ITT) basis using the full set of available data (mixed model and multiple imputation of missing data). Patients self-referring or being GP-referred receive equitable treatment, for example, in the length of wait to first physiotherapy contact once they are logged on the physiotherapy administration system. The physiotherapists participating in the trial will provide physiotherapy care to patients from both arms of the trial (initiated from traditional GP referral in the control arm versus from both traditional GP referral and patient self-referral in the intervention arm). As this trial is investigating the addition of direct access to physiotherapy, the physiotherapy care provided will be determined by clinical need and assessment findings and will therefore be consistent with routine practice and not differ for patients in different arms of the trial.

### Description of intervention and control arms

Control practices: In GP practices allocated to the control arm patients will continue to be managed according to usual GP-led care. This normally consists of a patient consulting their GP and receiving advice and treatment (often medication but we expect around 20–25 % to be referred to physiotherapy, or for a diagnostic test or other treatment service). For some patients, it may mean being triaged by a practice nurse and managed with advice and/or referral to physiotherapy or other services. Usual GP-led care therefore includes all of these pathways in participating GP practices but will not include the offer of direct access to physiotherapy. No additional information on physiotherapy services will be disseminated by the research team to GP practices delivering usual care. Regular contact with, and feedback to, the control practices through CRN staff, a GP research facilitator and from the PI and study team will help ensure smooth trial operationalisation.

Intervention practices: In practices allocated to the intervention arm patients will continue to be able to access care via usual GP-led care pathways (as described above) in addition to direct access to physiotherapy for musculoskeletal patients. Direct access will allow adults with musculoskeletal problems to refer themselves to the physiotherapy service. Those opting for direct access to physiotherapy will complete a self-referral form, which will be available at their GP practice or, where possible, online. The self-referral form will include questions about ‘red flag’ symptoms, indicative of suspected serious pathology that would require urgent medical investigation, with advice to patients to contact their GP if one or more of these symptoms are present. Submitted self-referral forms will be reviewed by a senior physiotherapist who will identify, based on existing physiotherapy service criteria, whether the referral is appropriate and whether classed as urgent or routine. Urgent cases will be offered the next available appointment whereas routine cases will join all other routine patients on a waiting list for the physiotherapy service and be seen in order of receipt of referral. In cases where the referral is considered inappropriate for physiotherapy management, the patient will be contacted by the physiotherapy service and signposted to the most appropriate practitioner or service. Direct access to physiotherapy will be actively marketed in the intervention practices to ensure that all registered patients are aware of this option [30]. This will start eight weeks prior to participant recruitment to the research evaluation and continue until patient recruitment to the trial has finished. Marketing will include communications with practice staff (clinical and administrative teams), practice posters, rolling presentations in practice waiting areas (where possible), patient flyers and self-referral forms in practices. In order to best ensure that all adults in the practice become aware of the availability of direct access, an information letter and direct access flyer will be mailed to all registered adult patients at each intervention practice. A phased approach to the marketing of the new service is planned, over 8 weeks, so that demand for physiotherapy through direct access can be monitored. The posters, flyers and forms will clearly explain how patients can directly access the service. The physiotherapy administrator will input patients’ data into the trial registration database and be responsible for arranging face-to-face physiotherapy appointments at convenient times and dates following prioritisation of the referrals by senior physiotherapists in the service. Even with the offer of direct access, we anticipate that many patients will opt to consult their GP as usual. Therefore, in the intervention practices, patients may consult their practice and be referred to physiotherapy, either by written referral or be recommended to access physiotherapy by GPs or nurse practitioners (GP or nurse recommended self-referral) or choose to directly access physiotherapy without contacting their GP practice (true self-referral). Each patient’s pathway will be recorded.

### Support for physiotherapists providing the direct access service

Although physiotherapists are autonomous practitioners on qualification, additional education and development needs of physiotherapists have been identified as part of the work-up of the pilot trial and a training programme is planned to address these. The brief training programme will include the following content: prevalence and identification of red flags, serious pathology and medical masqueraders, review of common over-the-counter and prescribed medications for musculoskeletal pain patients, the provision of occupational/work advice to patients and information about the practicalities of the trial, including delivery of the trial protocols and identification of any serious pathologies or serious adverse events. Continuing support of physiotherapists delivering the direct access service will take the form of a mentoring programme provided by the lead musculoskeletal physiotherapist in the team and another senior physiotherapist who has experience of providing direct access services. The physiotherapy service leads will also agree pathways for patients using direct access with suspected serious pathology who are in need of medical attention, or who may require a fit note or a prescription from their GP. Approximately 18 physiotherapists will participate in the training programme, half of whom will initially provide the new direct access physiotherapy service and the remainder will be able to provide cover as needed throughout the duration of the trial, for example for holiday periods.

### Outcome measures

Process outcomes: Process outcomes will determine if a future main trial is possible and desirable. Anonymised process outcome data will be collected from GP records and physiotherapy administration databases and self-referral forms. Health service process outcomes include the proportion of patients not referred to physiotherapy services, those referred to physiotherapy and the number of patients directly accessing physiotherapy in the intervention practices, proportion of ‘recommended self-referral’ and ‘true self-referral’ in the direct access pathway, waiting times for treatment (time from logging of referral to first physiotherapy appointment), number of GP and NHS physiotherapy consultations for musculoskeletal problems and non-attended physiotherapy appointments (DNA rates). Research process outcomes include estimation of recruitment rates, exploration of evidence of selection bias and follow-up rates at each time-point with differences in the control and intervention practices explored to identify sources of bias. A summary of the process measures is shown in Table 1.

**Table 1:** STEMS pilot trial process measures

Engagement of GP practices and PT services
Physiotherapy site recruitment rate to participate in STEMS study
GP Practice recruitment rate to participate in STEMS study
Research evaluation
Recruitment rate to the research evaluation—all adults with musculoskeletal conditions
Recruitment rate to the research evaluation—‘true self-referral’^a^ patients
Recruitment rate to the research evaluation—‘recommended self-referral’^b^ patients
Retention rates to research evaluation at 2, 6 and 12 months follow-up
GP practice characteristics
Number of GPs and nurse practitioners per practice
Number of patients and number of adults registered at practice
GP consultation rates for adults with musculoskeletal conditions (before and during study)
Physiotherapy team characteristics
Number of physiotherapists working in the physiotherapy service (in total and STEMS trained)
Seniority of physiotherapists in the physiotherapy service (in total and STEMS trained)
Physiotherapy service
Physiotherapy service GP referral rate (before and during study)
Physiotherapy service ‘true self-referral’^a^ rate
Physiotherapy service ‘recommended self-referral’^b^ rate
Non-attendance rates at physiotherapy site for GP referrals (before and during study) and for self-referrals during study
Number of physiotherapy consultations for GP referrals, ‘true self-referral’ and ‘recommended self-referral’ patients in the intervention practices
Number of self-referring patients deemed unsuitable at each stage in the direct access pathway
Onward referral rate from physiotherapy (to GP, other services) for GP referrals, ‘true self-referral’ and ‘recommended self-referral’
Physiotherapy waiting time (month-by-month) from 12 months prior to introduction of direct access to end of study
Number of patient complaints about direct access at physiotherapy site
Monitoring of safety
Number of cases of missed serious pathology in patients directly accessing physiotherapy
Number of adverse events in GP-referred, ‘true self-referral’ ‘recommended self-referral’

^a^Those who are prompted by their GP or practice nurse to access physiotherapy care

^b^Those who directly access physiotherapy care without prompting by their GP or practice nurse

Clinical outcomes: Data collection will include the collection of clinical and cost data through patient self-report questionnaires at baseline and 2, 6 and 12 months follow-up. The primary clinical outcome is physical health measured using the SF36v2 Physical Component Summary. A summary of the clinical outcomes and other measures included in each questionnaire is shown in Table 2.

**Table 2:** STEMS pilot trial questionnaire measures

Domains	Description				
		Baseline	2 months	6 months	12 months
Primary outcome measure					
Physical function	SF36v2 physical component summary http://www.sf-36.org/	✓	✓	✓	✓
Secondary outcome measures					
Overall change in condition	Global assessment of change since baseline—single question	✘	✓	✓	✓
Mental health	SF36v2 mental component summary http://www.sf-36.org/	✓	✓	✓	✓
Quality of life	EuroQol EQ-5D-5 L www.euroqol.org	✓	✓	✓	✓
Self-efficacy	Pain self-efficacy questionnaire (PSEQ) [31]	✓	✓	✓	✓
Understanding of condition	General practice assessment questionnaire enablement subscale [32]	✓	✓	✓	✘
Experience of consultations	General practice assessment questionnaire communication [32]	✓	✓	✓	✘
Accessibility of services	Single question	✘	✓	✓	✘
Satisfaction with services	Single question	✘	✓	✓	✘
Baseline measures					
Demographics	Gender, date of birth, ethnicity, education, health literacy, employment status, socio-economic status (recent paid job title, housing)	✓	✘	✘	✘
Baseline risk of persistent problems	STarT Musc tool (draft tool developed at Keele University to identify patients’ risk of persistent pain and disability)	✓	✘	✘	✘
Pain location	Body manikin	✓	✘	✘	✘
Pain duration	Single question about duration of pain	✓	✘	✘	✘
Comorbidities	Single question	✓	✘	✘	✘
Economic outcomes					
Further health care utilisation	Consultations, investigations, procedures, admissions, over-the-counter medications	✘	✘	✓	✓
Work absence	Single question	✓	✓	✓	✓
Presenteeism	Single work performance question	✓	✓	✓	✓
Willingness to pay	Three willingness to pay questions	✘	✘	✘	✓

### Adverse events

The occurrence of adverse events from all interventions will be monitored and assessed using case report forms, contact with the trial coordinator, physiotherapist report, and follow-up questionnaires. However, as the intervention is the introduction of a direct access pathway and the treatments received will be the same as in usual clinical practice, adverse events are unlikely. Physiotherapists and GPs will report any serious adverse event (SAE) experienced by a trial participant immediately to the trial chief investigator that may possibly be related to either the interventions or the trial procedures. The chief investigator will assess whether the event was related to or resulted from any of the STEMS study trial interventions or procedures. Any SAE considered to be related to the trial procedures or interventions will be reported to the main Research Ethics Committee by the chief investigator within 15 days of her becoming aware of the event. In addition, all such events will be reported to the trial sponsor, Trial Steering Committee and Data Monitoring Committee.

### Safety of direct access to physiotherapy

Any serious or significant pathologies that would require urgent medical assessment missed by physiotherapists in patients who directly access care will be reported. Physiotherapists and GPs will be asked to report any cases they become aware of. In addition, a systematic search of the GP medical record for participants who consent to medical record review and who directly access physiotherapy care will be undertaken to identify any possible cases of missed serious or significant pathology. The number of missed serious pathologies will be described only for participants directly accessing physiotherapy.

### Sample size

As this is a pilot trial, a formal sample size calculation has not been carried out. Using Keele’s Consultation in Primary Care Archive (CiPCA) database of GP practices and symptom and diagnostic Read codes, 25 % of the population in an average-sized GP practice (5000 patients) consults at least once per year with a musculoskeletal problem (1250 patients or 100 patients per month). Given the trial exclusions, we anticipate at least 80 % of consulters to be eligible (80 patients per month per practice), 50 % to respond to the invitation to take part in the research evaluation, provide consent and complete a baseline questionnaire (40 patients per month per practice). With four practices recruiting for 6 months in the pilot trial, we estimate 960 participants at baseline and 80 % follow-up (*n* = 768).

### Analysis

Since this is a pilot trial, the analyses will focus on describing the key process measures in order to decide if a main trial is feasible and desirable, in addition to finalising the sample size for a future main trial.

#### Determine engagement of GP practices and the physiotherapy service

The number of GP practices and physiotherapy services that have agreed to participate in the trial will be recorded and presented as a proportion of the number of eligible practices approached. Information will be presented that summarises GP practice characteristics (full-time equivalent of GP staff, number of patients and number of adults registered at practice and GP consultation rates for musculoskeletal patients before and during pilot trial recruitment as well as the physiotherapy team characteristics (full-time equivalent staff and their clinical grades).

#### Feasibility of study recruitment and retention

The number of participants identified and recruited using each recruitment method will be reported, along with the number of participants followed up at each time-point. Withdrawals (and where possible, reasons for withdrawals) will be reported. A priori, we have defined a success criterion of 40 % of the total number of participants invited to be recruited to the research evaluation. Whilst a retention rate of 100 % would be ideal, we will consider a rate of 70 % at 6 months follow-up satisfactory. We will provide the point estimate of the proportion and its 95 % confidence interval (CI). Difference in recruitment uptake rate and follow-up rates at each time-point will be compared between the intervention and control arms.

#### Feasibility of direct access

An audit of the feasibility of offering direct access will be conducted using anonymised data. This will include reporting the number of participants who are referred to physiotherapy by their GP, those who are ‘recommended self-referrals’ (prompted by their GP or practice nurse to refer) and those who are ‘true self-referrals’ and the waiting time to the first physiotherapy appointment. Figures will be presented month-by-month for patients using direct access as well as the overall totals. The proportion of patients directly accessing care (true and recommended self-referrals) compared to all referrals received at the physiotherapy site will be calculated to check the success of marketing the direct access service. The aim is to ensure that a minimum of 20 % of the total physiotherapy caseload during the pilot trial, from practices randomised to the intervention arm, is through direct access. In order to assess the feasibility of establishing a direct access service that can respond to demand, descriptive measures of the total number of referrals received from participating practices and staffing levels in the participating physiotherapy department will be used to establish how the physiotherapy service responds to demand through waiting time for first physiotherapy appointment. The source by which patients, who access care directly, become aware of the direct access service will be presented and compared in order to establish which methods (GP advised, poster, individual mailing, heard from a friend and other methods) are most successful in marketing the service.

#### Evidence of selection bias

Since this is a pilot trial with only four clusters randomised, it is likely that there will be some imbalance between participants in each of the treatment arms on one or more baseline characteristics. Baseline comparisons will be carried out to detect any substantial differences between participants recruited from the control and intervention arms. This will be done by scrutinising the baseline table for any serious imbalances in observable baseline variables and the trends of the imbalance if any. The recruitment rates will also be estimated and compared between the control and intervention arms. We will examine the size of any imbalances and decide if there is evidence of systematic selection bias in the types of patients being recruited in control versus intervention arms. Any systematic imbalance in the sense that one arm is consistently favoured by the imbalances may not reflect chance alone and may suggest selection bias.

#### Explore generalisability of the sample

Anonymised data on key baseline characteristics (age, gender and index of multiple deprivation (from post-codes)) of those who are invited but who do not participate will be compared with those who do participate. Key baseline characteristics will be compared between those participants followed up and those lost to follow-up at each time-point. Key characteristics of the participants recruited using each recruitment method will be reported. Delays in return of questionnaires (>35 days from initial mailing date) will also be compared between the two arms.

#### Analyses of clinical outcomes

Analyses will be conducted for the clinical outcomes, but this will be treated as exploratory and will be mainly descriptive. A baseline table (descriptive statistics and frequencies) will compare the demographic and clinical characteristics (gender, age, education, employment status, pain interference with performance at work, type of accommodation, health literacy, physical health (SF-36v2 PCS) and mental health (SF-36v2 MCS), pain location, physical function, pain duration, baseline risk of persistent problems, comorbidities, pain self-efficacy, understanding of the condition, experience of care and convenience and accessibility of service) between the two arms. Since the baseline assessment for the clinical characteristics will be completed, for many patients, after their initial consultation about their musculoskeletal problem, there could be implications on evaluation and interpretation of these measures as they may not be ‘true baseline’ but rather possible ‘outcomes’ at the first time-point. All continuous variables will be summarised using mean, standard deviation, median and interquartile range as appropriate. The frequency and percentages of observed levels will be reported for all categorical measures.

As this is a pilot trial, no emphasis will be put on the *p* values for any inferential statistical tests conducted. A mixed effect model, which allows all available data at all the four time-points to be used and account for missing data and clustering effect, will be used to estimate a two-sided 95 % CI to show a credible range for the true difference in the SF-36v2 PCS subscale between intervention and the control arms. The model will be adjusted for key patient-level baseline characteristics (age, area level deprivation and widespread pain (from manikin data)) and a random effect for the GP practices and will include a treatment-by-time interaction to obtain the estimates of treatment effect (and 95% CI) at each follow-up visit (2, 6 and 12 months). Analyses of the secondary outcomes will be performed similarly.

Further analysis will descriptively compare the baseline characteristics and key clinical outcomes between the participants referred to physiotherapy in the control practices with participants self-referring in the intervention practices. We will also compare the self-referrers with those that do not self-refer within the intervention group.

The pilot data will provide information on the parameters needed for a realistic sample size calculation (mean, standard deviation and treatment effects of the primary outcome for the two arms) for a future, main cluster RCT. The intra-cluster correlation (ICC) will be calculated and compared with the estimates from previous large primary-care-based trials. However, as we only have 4 GP practices, we expect the estimated ICC to be unreliable given the likely wide confidence intervals. The non-inferiority margin will not be calculated from the pilot data as previous research in musculoskeletal disorders has estimated a minimal clinically important difference (MCID) from 2 to 4 points for the SF-36v2 Physical Component Summary (PCS) subscale which will be used to inform the non-inferiority margin of any future main trial [33–36].

#### Missing outcome data

Since the primary outcome measure, physical health measured using the SF-36v2 PCS involves scoring individual items, we do not expect all returned questionnaires to have every item on the SF-36 completed. Based on the recommendation in the scoring manual [33], a scale score should be calculated if a respondent answers at least 50 % of the items in a scale. Missing data will be estimated by the average score, across completed items in the same scale for that respondent. If more than 50 % of the items in a scale are left blank, the imputation algorithm identifies the scale as ‘not computable’ and so the respondent is considered to have missing data for the SF-36v2 component scales.

In order to explore the extent and patterns of missing outcome data, we will report the proportion of missing values per item, proportion of participants who complete all items on the questionnaire and the proportion of respondents who answer at least 50 % of the items in a scale. The proportion of missing data will also be reported for the other key outcomes and compared between the participants from intervention and control practices. The characteristics of those lost to follow-up will also be compared with those who remain in the trial through follow-up.

#### Safety of direct access

Any possible cases of missed serious or significant pathology will be discussed within the study team. For any patient where there is evidence to suggest the physiotherapist failed to correctly identify this pathology when the patient directly accessed physiotherapy care and medical intervention was delayed will be reported.

#### Health economic analysis

The economic analysis will be exploratory, with the aim to inform the design of a full cost-utility analysis alongside a future main trial. A cost-consequence analysis will be reported, describing all the important results relating to costs and consequences for direct access to physiotherapy and usual care arms of the trial. Results will be presented from a health service perspective and a broader societal perspective taking into account patient-incurred costs and productivity losses. An exploratory willingness to pay (WTP) study [37] will also be conducted to assess the use of this methodology to measure broader benefits of direct access.

Data on costs will be sought from all participants and from a broad perspective, taking into account healthcare, patient and societal costs. Healthcare resource used will be collected using self-completed questionnaires at 6 and 12 months, with a recall period of 6 months in each. Questions will ask patients to recall GP consultations, visits to healthcare professionals, outpatient appointments, investigations or treatments and inpatient stays related to the index condition. Participants will be asked to distinguish between NHS and private practice visits. Resource used for the direct access pathway will be directly recorded and costs attached, staff time (taking into account any increased referrals to physiotherapy), materials (posters, flyers, referral forms) and training sessions. GP medical record review of all participants will provide data on relevant prescribed medications for all musculoskeletal problems. In addition, information on costs borne by the patient (e.g. over-the-counter medicines, devices) will be collected via the self-complete questionnaires. Productivity costs will take into account both absenteeism and presenteeism and will utilise self-report data on employment status, occupation and time off work and reduced productivity at work (presenteeism). As this is a pilot trial, the suitability of questions for collecting cost data directly from patients can be assessed, in order to ensure effective resource use and that cost data collection systems are established for a larger trial.

All patients will be asked to complete the 5-level version of the EuroQoL-5D (EQ-5D) questionnaire at baseline, 6 months and 12 months in order for the quality-adjusted life years (QALYs) over the 12-month time period to be calculated for each participant. The QALYs combine information on health-related quality of life and survival.

Resource use will be multiplied by unit costs obtained from standard sources (NHS Reference Costs, British National Formulary, Unit Costs of Health and Social Care) and healthcare providers. Due to the lack of nationally representative unit cost estimates for private healthcare, this care will be costed as the NHS equivalent in the base case. Patient reported costs for over-the-counter treatments will be used. Productivity costs will be calculated using data collected on employment status at every time-point and days off work due to their musculoskeletal problem. For those in paid employment, information on occupation and the nature of their employment (full time or part time) will be requested. The average wage for each respondent will be identified using UK Standard Occupational Classification coding and annual earnings data for each job type. The analysis will use the human capital approach, and the self-reported days of absence will be multiplied by the respondent-specific wage rate. The human capital approach assumes that the value of lost work is equal to the amount of resources an individual would have been paid to do that work and values productivity losses as a result of morbidity (or mortality) by measuring time lost from work and multiplying this with the gross wage of the person. Responses to the EQ-5D questionnaire at baseline and 6 and 12 months will be used to calculate QALYs for each participant using the area under the curve method.

As this is a pilot trial this will be an exploratory analysis, with the aim to inform the cost data collection and analysis of a future main larger trial. We will test our methods of collecting cost data, from patient questionnaires and medical record reviews and assess the completeness of the data collected. A cost-consequence analysis will be reported, describing all the important results relating to costs and consequences. Analyses will be mainly descriptive, and all costs and outcomes will be summarised using means and 95 % confidence intervals. The data for costs are likely to have a skewed distribution; therefore, the plan is to explore the nature of the distribution of costs. If the data is not normally distributed, a non-parametric comparison of means (e.g. bootstrapping) will be undertaken.

The base case cost analysis will adopt a NHS and personal social services (PSS) perspective. A broader costing perspective will be considered in a sensitivity analysis, taking into account NHS/PSS costs, patients’ personal expenditure and costs associated with work loss. The robustness of the results will be explored using sensitivity analysis and will explore uncertainties in the trial based data and any assumptions made in the base case analysis.

##### Nested willingness to pay analysis

Exploratory work using a willingness to pay (WTP) approach that fits within a cost-benefit analysis framework will also be conducted in this pilot trial. Cost-benefit analysis is ideally placed for the evaluation of interventions where the benefits may be non-health related. The approach is one way of measuring how valuable a service is and how much (in monetary terms) participants would be willing to give up in order to receive it. The hypothetical nature of the question is highlighted in the information given to participants. The WTP study questionnaire will be sent to all participants at 12 months. The main WTP question contains presenting the participant with a range of monetary values in a table from £0 to £350 and asks participants what is the maximum amount of money they would be willing to pay to have direct access to a physiotherapy service. A space is provided for them to explain the reasons for their answer, and there are further questions asking if they would use a direct access service if available and their annual household income.

Firstly, the pilot study will test the completion rate of the WTP questions and whether WTP can be measured in this context. This does not aim to obtain definitive WTP findings but to explore the feasibility of the method for use in a future main trial. Descriptive statistics for willingness to pay values will be presented in both trial arms, including the proportion of protest zero responses. ‘Protest zeros’ occur when participants report a zero WTP even though they value the service (in contrast to reporting a zero WTP if they do not value the service) and are usually due to refusal to engage with the WTP questions. All reasons for responses will be coded. A preliminary linear regression analysis will be undertaken to explore the relationship between stated WTP and participant characteristics, using WTP values as the dependent variable and variables such as trial arm, age, gender, health status and income as independent variables.

### Trial organisation and monitoring

The STEMS pilot trial is sponsored by Keele University. The day-to-day operation of the trial will be overseen by a Trial Management Group (led by AB), in line with the Standard Operating Procedures of the Keele Clinical Trials Unit. The Trial Management Group will meet monthly and AB will meet more frequently with the study coordinator. The trial will be monitored by an independent Trial Steering Committee (TSC) chaired by Professor Tracey Howe, who will meet approximately twice a year timed to coincide with key milestones, such as approval of the protocol, approval of the statistical analysis plan and interpretation of results. The TSC is made up of individuals with expertise in musculoskeletal research, delivery of care in general practice and lay members with musculoskeletal pain. An independent Data Monitoring Committee (DMC) monitors all trials conducted at the Research Institute for Primary Care and Health Sciences. Having reviewed the protocol and in view of the feasibility and pilot nature of the STEMS pilot trial, the DMC (chaired by Dr Janine Gray) proposed that monitoring of this study remains the responsibility of the TSC. During the pilot trial period no interim analyses are planned.

### Dissemination

Results from the STEMS pilot study will be disseminated through oral and poster presentations at conferences along with publications in peer review journals and other media. A report to the funder is a requirement of funding and will be submitted at the end of the study. Results will also be disseminated to participating general practices and the physiotherapy service through face-to-face meetings and/or electronic methods, depending on preference. Results will be made available on the Research Institute’s website and dissemination to participants will be coordinated in liaison with the Patient and Public Involvement coordinator. Keele University CTU has established data sharing arrangements to support joint publications and other research collaborations.

### Data confidentiality and archiving

The transportation of electronic sensitive data originating from NHS sources, such as contact details or medical records, will be conducted in accordance with the guidelines provided by NHS National Information Governance Board (NIGB). Data collected as part of the medical record review of consenting participants will be recorded on NHS compatible encrypted laptop computers and then transferred to university computers. Any transfer of personal information between CRN staff and GP practice or physiotherapy service administrators will be transferred using NHS email accounts. Paper records kept for research purposes will include hard copies of the completed questionnaires with signed consent forms and case report forms (CRFs). Consent forms will contain names and address but will be stored in a secure environment separate from patient data. The contact details of consenting participants will be required for mailing the follow-up questionnaires for this study. These contact details will be stored on a separate database to their questionnaire responses and clinical data, linked by their unique ID number. All trial-related information will be stored securely at the Research Institute for Primary Care and Health Sciences at Keele University. Coded identification numbers will be used to anonymise data with the data and the linking code stored in separate locations, under password protection. Access to the data will be to the small number of individuals necessary for quality control, audit and analysis. The final trial dataset will be accessed by the statistician (RO), the trial principal investigator (AB) and chief investigator (NEF). We will publish and communicate the pilot trial results regardless of the outcome of the trial. Data from the STEMS pilot trial will be archived and made available for future, secondary analysis and data pooling purposes from the Research Institute for Primary Care and Health Sciences at Keele University.

### Ethical review and trial registration

The STEMS pilot trial received research ethical approval from NRES Committee North West—Preston in February 2013 (REC reference, 13/NW/0053), and site-specific approvals have been received from the appropriate local research and development offices. The trial is being conducted in accordance with the ethical principles in the Declaration of Helsinki and good practice guidelines on the proper conduct of research. The STEMS pilot trial is registered at Current Controlled Trials ISRCTN23378642.

## Results and discussion

The STEMS pilot trial will investigate the feasibility of a future large trial to compare the clinical and cost-effectiveness of the additional offer of direct access to physiotherapy versus continuing with usual GP-led primary care alone for adults with common musculoskeletal problems. It is essential that changes to service provision and delivery are supported by research evidence and arguable that research about how to improve the delivery of health care is just as important in improving the health of patients with musculoskeletal conditions as the testing of new drugs or therapeutic approaches.

Previous research into direct access to physiotherapy has been limited to observational studies with the inherent limitations of observational designs. No previous trials have been conducted into direct access to physiotherapy for patients with musculoskeletal problems and this pilot trial will inform a main trial to fill this evidence gap and meet the calls to provide robust trial evidence of the impact on clinical outcomes, work loss and costs in a trial comparing areas that do and do not offer direct access [26] and to provide clinicians and service commissioners with the high quality trial data they need to guide decisions on the best models of care [25]. Direct access will be deemed to be the preferred model if it is not inferior in terms of patients’ physical health (on the SF-36v2 PCS—the main trial primary outcome) and is associated with benefits such as shorter waiting times, greater satisfaction, improved work outcomes and greater cost-effectiveness. Direct access may prove to be more cost-effective if it reduces other health service consultations and prescriptions, or improves quality of life or less cost-effective if it involves additional physiotherapy consultations with no reduction in GP consultations or prescriptions or improvements in quality of life

The strengths of the STEMS pilot trial are its size, the length of follow-up and collection of process, clinical and cost outcomes to fully inform a future main trial. In addition, marketing of the direct access service at GP practice level will avoid contamination between arms but will ensure that all registered adult patients are aware of the direct access service. This will enable sufficient participants to directly access physiotherapy care during the pilot trial to allow planned analyses. The care pathway to physiotherapy in the intervention practices will be set up to mimic closely what would happen if direct access were routinely available. Although patients in the intervention arm may choose to access physiotherapy directly without ever contacting their general practice about their musculoskeletal condition (true self-referrers), some patients will contact their practice and may be advised that they can self-refer to physiotherapy (recommended self-referrers). The facility for GPs to send a written referral in the traditional manner also continues as this may be particularly suitable for some patients e.g. the frail elderly or those with very complex problems.

### Design considerations and limitations

We acknowledge that a limitation of the trial design is recruiting patients in the control arm from GP practices and in the intervention arm from GP practices and the physiotherapy service. In an ideal design, we would compare all patients who have a musculoskeletal problem registered with participating practices during the study period, whether or not they attend their GP and/or physiotherapy service but clearly this is not possible. Other trial designs were considered, but these would have led to greater differences in patients recruited to the two arms of the trial. We will therefore make considerable efforts to identify any evidence of selection and recruitment bias between the arms of the pilot trial, although we acknowledge that patients may differ on other unknown factors.

It is possible that over time, as patients become accustomed to direct access, their expectations and service use may change. Clinical outcomes are unlikely to change beyond 12-month follow-ups, but costs and benefits may change as direct access becomes established within a community. Therefore, this pilot trial includes follow-up to 12 months as longer follow-up would be expensive within the context of a trial.

This pilot aims to inform a main trial with a non-inferiority design as the addition of a direct access to physiotherapy pathway is not anticipated to be superior in terms of patients’ clinical outcomes. However, a range of secondary outcomes is included that in a main trial will provide important information for patients, health care practitioners and commissioners if clinical non-inferiority is demonstrated. Previous work suggests an effect size of 0.2 to 0.6 as a minimum clinically important difference, and we would thus, anticipate specifying a difference between groups of 0.2 as the threshold for the purposes of demonstrating non-inferiority in a main trial.

## Conclusions

This pilot cluster randomised control trial will provide valuable information to inform a future future large trial to compare the clinical and cost-effectiveness of the additional offer of direct access to physiotherapy versus continuing with usual GP-led primary care alone for adults with common musculoskeletal problems

## Additional file

Additional file 1:
**STEMS Participant Information Leaflet.**

## Abbreviations

CI: confidence Interval
CiPCA: Consultation in primary care archive
CRN: Clinical Research Network
CSP: Chartered Society of Physiotherapy
DMC: Data Monitoring Committee
DNA: did not attend
DoH: Department of Health
GP: general practitioner
ICC: inter-cluster correlation
ITT: intention to treat
NHS: National Health Service
NRES: National Research Ethics Service
PCS: Physical Component Summary
PSS: personal social services
QALY: quality-adjusted life year
QIPP: quality and productivity case studies
RCT: randomized controlled trial
SAE: serious adverse event
SF36v2: short form 36 version 2
STEMS: Stepping up the evidence for musculoskeletal services
TSC: Trial Steering Committee
UK: United Kingdom
US: United States
WTP: willingness to pay

## Footnotes

1 Read codes are the standard diagnostic codes used in general practice in the UK
