# Supplementary material for: A pilot cluster randomised controlled trial to investigate the addition of direct access to physiotherapy to usual GP-led primary care for adults with musculoskeletal pain: the STEMS pilot trial protocol (ISRCTN23378642)
Source: Pilot Feasibility Stud. 2015 Jul 17;1:26. doi: 10.1186/s40814-015-0020-4 (PMC5154068; doi:10.1186/s40814-015-0020-4)
Supplement: Additional file 1: — STEMS Participant Information Leaflet. [file 40814_2015_20_MOESM1_ESM.pdf]

What if something goes wrong?

If you wish to complain about any aspect of the way you have been approached or treated during the course of this study, the normal National Health Service (NHS) complaints procedure will be available to you. Further details of this are available from your GP practice.

What will happen to the results of the study?

Once the study is completed we intend that the results will be published in a medical journal and shared within the NHS. A summary of results will be sent to all participants.

Who is organising and funding the study?

The research is organised by the Arthritis Research UK Primary Care Centre at Keele University along with your GP practice and local physiotherapy services. The research has been funded by the Charitable Trust of the Chartered Society of Physiotherapy.

Who has reviewed the study?

The NRES Committee North West – Preston, Research Ethics Committee has reviewed this study (Research Ethics Committee Reference Number: 13/NW/0053.

Contact for further information

If you have any questions, or would like further information, about this study please contact our Study Coordinator, Stephanie Tooth on 01782 733921.

If you would like help competing the questionnaire, please contact Julie Young our research nurse on 01782 733921.

If you have any questions or concerns about taking part in this research you can also contact the Patient Advice and Liaison Service (PALS) on 01625 661449.

Thank you for taking the time to read this leaflet

Arthritis Research UK Primary Care Centre

Primary Care Sciences

Keele University

Staffordshire, ST5 5BG

Tel: +44 (0) 1782 733921

Fax: +44 (0) 1782 733911

Arthritis Research UK

Providing answers today and tomorrow

Keele University

STepping up the Evidence for Musculoskeletal Services

STEMS Study

Patient Information Leaflet

You are being asked to take part in a research study called STEMS. Your GP practice and physiotherapy service are working with the research team at the Arthritis Research UK Primary Care Centre at Keele University to learn more about services available for patients with pain or pain related symptoms. By pain related symptoms we mean aches, pain, discomfort, tingling or stiffness. Please take time to read this information carefully and discuss it with others (such as friends and family) if you wish. It is important to us that you understand why the research is being done and what it involves.

What is the purpose of the study?

This study is evaluating local NHS services for pain conditions. Aches and pains are very common symptoms. We are trying to find out more about your pain or pain related symptoms and the services available to help you.

We would like to gather information in 2 ways:

1) With questionnaires, the first one is included in this pack and three more will be sent to you over the next year to track any changes in your condition, and

2) By looking at your medical records.

Why have I been chosen?

You were chosen because you have recently contacted a health care professional (e.g. GP, Nurse, Physiotherapist) with pain or pain related symptoms.

What might be the risks of taking part?

We do not expect any problems for you from taking part in this study.

What are the benefits of taking part?

Although there is no direct benefit from taking part in this study, what we learn from the study should help us develop future services for patients like you.

What if my symptoms are already better?

It is valuable for us to have information about you even if you don't have pain at the moment.

Will my taking part in this study be kept confidential?

All the information you give us will be treated in the strictest confidence. Each person who takes part in this study will be given a code number, so the data from the study will not have any identifiable names and addresses, and cannot be traced back to you. On this basis, the data will be anonymous and may be used in other research studies.

Do I have to take part?

Taking part in the STEMS study is purely voluntary and if you choose not to take part in this study your medical treatment will not be affected in any way. You may also withdraw from the study at any time without this affecting your future care. If you wish to take part, you will need to sign the consent form at the end of the first questionnaire (included with this information sheet).

If you choose not to take part, you don't have to tell anybody why and you are free to withdraw from the study at any time, even after you have signed the consent form.

What will happen if I take part?

This study does not affect the health care you receive. There are no additional clinic visits or tests involved. The study involves four questionnaires in total over a period of 12 months and a medical record review, where a researcher will collect information from your medical records about your pain or related symptoms over the 12 months of the study.

1) Questionnaire: by asking you to complete 4 questionnaires spread out over the next year, we hope to find out more about what happens to people with pain or pain related symptoms. The first questionnaire and consent form is included in this pack. We will then send you further questionnaires; one in 2 months, one in 6 months and one in 12 months' time. These questionnaires are completed by you at home. The questionnaires ask about your pain or related symptoms, your general health and any services or care you have received. Each questionnaire should take approximately 30 minutes to complete.

2) Medical record review: Your name will not be linked with any information taken from your medical records once the review has been carried out.
